# Cognitive trio: relationship with major depression and clinical predictors in Han Chinese women

**DOI:** 10.1017/S0033291713000160

**Published:** 2013-02-21

**Authors:** L. Wang, L. Liu, S. Shi, J. Gao, Y. Liu, Y. Li, Z. Zhang, G. Wang, K. Zhang, M. Tao, C. Gao, K. Li, X. Wang, L. Lv, G. Jiang, X. Wang, H. Jia, J. Zhang, C. Lu, Y. Li, K. Li, C. Hu, Y. Ning, Y. Li, J. Sun, T. Liu, Y. Zhang, B. Ha, H. Tian, H. Meng, J. Hu, Y. Chen, H. Deng, G. Huang, W. Wu, G. Li, X. Fang, J. Pan, X. Hong, S. Gao, X. Li, D. Yang, G. Chen, T. Liu, M. Cai, J. Dong, Q. Mei, Z. Shen, R. Pan, Z. Liu, X. Wang, Y. Tan, J. Flint, K. S. Kendler

**Affiliations:** 1 Shandong Mental Health Center, Jinan, Shandong, P.R. China (P.R.C.); 2 Fudan University Affiliated Huashan Hospital, Shanghai, P.R.C.; 3 ZheJiang Traditional Chinese Medical Hospital, Hangzhou Zhe Jiang, P.R.C.; 4 The First Hospital of China Medical University, He Ping District, Shenyang, Liaoning, P.R.C.; 5 No. 1 Hospital of Zhengzhou University, Zhengzhou, Henan, P.R.C.; 6 No. 4 Affiliated Hospital of Jiangsu University, Zhenjiang, Jiangsu, P.R.C.; 7 Beijing Anding Hospital, Capital Medical University, Xicheng District, Beijing, P.R.C.; 8 No. 1 Hospital of Shanxi Medical University, Taiyuan, Shanxi, P.R.C.; 9 Second Affiliated Hospital of Zhejiang Chinese Medical University, Hangzhou, Zhe Jiang, P.R.C.; 10 No. 1 Hospital of Medical College of Xian Jiaotong University, Xi'an, Shaanxi, P.R.C.; 11 Mental Hospital of Jiangxi Province, Jiangxi, P.R.C.; 12 Shengjing Hospital of China Medical University, Heping District Shenyang, Liaoning, P.R.C.; 13 Psychiatric Hospital of Henan Province, Xinxiang, Henan, P.R.C.; 14 Chongqing Mental Health Center, Chongqing, P.R.C.; 15 The First Hospital of Hebei Medical University, Shijiazhuang, Hebei, P.R.C.; 16 Jilin Brain Hospital, Siping, Jilin, P.R.C.; 17 No. 3 Affiliated Hospital of Sun Yat-sen University, Tian He District, Guangzhou, Guangdong, P.R.C.; 18 Dalian No. 7 People's Hospital & Dalian Mental Health Center, Gan Jing Zi District, Dalian, Liaoning, P.R.C.; 19 Hebei Mental Health Center, Baoding, Hebei, P.R.C.; 20 No. 3 Hospital of Heilongjiang Province, Beian, Heilongjiang, P.R.C.; 21 Guangzhou Brain Hospital/Guangzhou Psychiatric Hospital, Fang Cun Da Dao, Li Wan District, Guangzhou, Guangdong, P.R.C.; 22 Wuhan Mental Health Center, Wuhan, P.R.C.; 23 Nanjing Brain Hospital, Nanjing, Jiangsu, P.R.C.; 24 Shenzhen Kangning Hospital, Luo Hu, Shenzhen, Guangdong, P.R.C.; 25 Lanzhou University Second Hospital, Second Clinical Medical College of Lanzhou University, Lanzhou, Gansu Province, P.R.C.; 26 No. 4 People's Hospital of Liaocheng, Liaocheng, Shandong, P.R.C.; 27 Tianjin Anding Hospital, Hexi District, Tianjin, P.R.C.; 28 No. 1 Hospital of Chongqing Medical University, Yu Zhong District, Chongqing, P.R.C.; 29 No. 1 Mental Health Center Affiliated Harbin Medical University, Nangang District, Harbin, Heilongjiang, P.R.C.; 30 The Fourth Military Medical University Affiliated Xijing Hospital, Xi'an, Shaanxi, P.R.C.; 31 Mental Health Center of West China Hospital of Sichuan University, Wu Hou District, Chengdu, Sichuan, P.R.C.; 32 Sichuan Mental Health Center, Mian Yang, Sichuan, P.R.C.; 33 Shanghai Tongji University Affiliated Tongji Hospital, Shanghai, P.R.C.; 34 Mental Health Institute of Jining Medical College, Dai Zhuang, Bei Jiao, Jining, Shandong, P.R.C.; 35 Fuzhou Psychiatric Hospital, Cang Shan District, Fuzhou, Fujian, P.R.C.; 36 The First Affiliated Hospital of Jinan University, Tian He District, Guangzhou, Guangdong, P.R.C.; 37 Mental Health Center of Shantou University, Wan Ji Industrial Zone, Tai Shan Bei Lu, Shantou, Guangdong, P.R.C.; 38 Ningbo Kang Ning Hospital, Zhen Hai District, Ningbo, P.R.C.; 39 Anhui Mental Health Center, Hefei, Anhui, P.R.C.; 40 Jining Psychiatric Hospital, Bei Dai Zhuang, Ren Cheng District, Jining, Shandong, P.R.C.; 41 Huaian No. 3 Hospital, Huaian, Jiangsu, P.R.C.; 42 No. 2 Xiangya Hospital of Zhongnan University, Changsha, Hunan, P.R.C.; 43 Huzhou No. 3 Hospital, Huzhou, Zhejiang, P.R.C.; 44 Qingdao Mental Health Center, Shibei District, Qingdao, Shandong, P.R.C.; 45 Suzhou Guangji Hospital, Suzhou, Jiangsu, P.R.C.; 46 Tangshan No. 5 Hospital, Lu Nan District, Tangshan, Hebei, P.R.C.; 47 Guangxi Longquanshan Hospital, Yu Feng District, Liuzhou, P.R.C.; 48 Anshan Psychiatric Rehabilitation Hospital, Li Shan District, Anshan, Liaoning, P.R.C.; 49 Renmin Hospital of Wuhan University, Wu Chang District, Wuhan, Hubei, P.R.C.; 50 Beijing Huilongguan Hospital, Changping District, Beijing, P.R.C.; 51 Wellcome Trust Centre for Human Genetics, Oxford, UK; 52 Virginia Institute for Psychiatric and Behavioral Genetics, Department of Psychiatry, Virginia Commonwealth University, Richmond, VA, USA

**Keywords:** Cognitive trio, Han Chinese women, major depression, suicide, symptoms

## Abstract

**Background:**

Previous studies support Beck's cognitive model of vulnerability to depression. However, the relationship between his cognitive triad and other clinical features and risk factors among those with major depression (MD) has rarely been systematically studied.

**Method:**

The three key cognitive symptoms of worthlessness, hopelessness and helplessness were assessed during their lifetime worst episode in 1970 Han Chinese women with recurrent MD. Diagnostic and other risk factor information was assessed at personal interview. Odds ratios (ORs) were calculated by logistic regression.

**Results:**

Compared to patients who did not endorse the cognitive trio, those who did had a greater number of DSM-IV A criteria, more individual depressive symptoms, an earlier age at onset, a greater number of episodes, and were more likely to meet diagnostic criteria for melancholia, postnatal depression, dysthymia and anxiety disorders. Hopelessness was highly related to all the suicidal symptomatology, with ORs ranging from 5.92 to 6.51. Neuroticism, stressful life events (SLEs) and a protective parental rearing style were associated with these cognitive symptoms.

**Conclusions:**

During the worst episode of MD in Han Chinese women, the endorsement of the cognitive trio was associated with a worse course of depression and an increased risk of suicide. Individuals with high levels of neuroticism, many SLEs and high parental protectiveness were at increased risk for these cognitive depressive symptoms. As in Western populations, symptoms of the cognitive trio appear to play a central role in the psychopathology of MD in Chinese women.

## Introduction

The etiological role of cognition in depression was first formulated by Beck (1963, 1967). In his theory, Beck proposed that depressive symptoms could be explained in cognitive terms resulting from the biased interpretations of events that are attributed to the activation of negative representations of the self, the personal world, and the future (the negative cognitive triad). The progressive dominance of these cognitive patterns leads to the affective and motivational symptoms that are associated with depression. According to Beck's cognitive diathesis-stress theory of depression (Beck, 1967, 1983), environmental or other stressors activate, through the individual's depressogenic schemata, the negative themes and cognitions that result in depressive illness (Ingram *et al.* 2006).

Substantial empirical evidence from both cross-sectional and longitudinal studies has supported Beck's theory that the interaction of cognitive vulnerability (e.g. dysfunctional attitudes) and life stress predicts depressive symptoms (e.g. Olinger *et al.* 1987; Abela & D'Alessandro, 2002; Hankin *et al.* 2004; Alloy *et al.* 2006*b*). For example, Abela & D'Alessandro (2002) found that scores on a youth measure of dysfunctional attitudes interacted with negative information about university admissions to predict increases in depressed mood over an 8-week period. These researchers examined two specific components of the negative triad and found that negative views of the future and the self predicted increases in depressed mood. In three longitudinal studies, Hankin *et al.* (2004) found that cognitive vulnerability interacted with negative life events to predict future depressive symptoms.

Several studies also provide evidence that cognitive vulnerability interacting with stressful life events (SLEs) predict the onset of major depression (MD) (Lewinsohn *et al.* 2001; Evans *et al.* 2005; Alloy *et al.* 2006*b*). Evans *et al.* (2005) examined the onset of MD in a large sample of 12 003 pregnant women. Compared with women who scored in the bottom third on a measure of schematic content (a measure pertaining to interpersonal sensitivity), those who scored in the upper third were more likely to suffer from depression within 3 years. In a 2.5-year longitudinal study, Alloy *et al.* (2006*b*) found that cognitively high-risk participants were 3.6–6.8 times more likely to experience MD than low-risk participants.

Cognitive behavioral therapy (CBT), which is based on Beck's cognitive model, is one of the most extensively applied forms of psychotherapy for MD. A recent review of 16 meta-analyses of CBT suggested that this therapy was highly effective for adult and adolescent unipolar depression (Butler *et al.* 2006). Results indicated that CBT was superior to antidepressants in the treatment of adult unipolar depression and produced significantly superior long-term persistence of effects with relapse rates half those of pharmacotherapy (DeRubeis *et al.* 2005). Segal *et al.* (1999) compared depressed patients who were successfully treated with either CBT or antidepressants and provided direct evidence that schema activation was related to depressive vulnerability. Participants were administered the Dysfunctional Attitudes Scale (DAS) when treated and were subsequently induced into a dysphoric mood state and then administered a parallel form of DAS. Individuals who received cognitive therapy did not show increase in negative thinking while individuals who were treated pharmacologically showed elevated DAS scores. Researchers also found that such changes in dysfunctional attitudes could predict relapse 18 months later (Segal *et al.* 2006). These findings together provided convincing evidence for Beck's theory of cognitive vulnerability to depression.

Despite the widely accepted efficacy of CBT as a treatment for depression, there are few studies of the relationship between the cognitive triad and the clinical features of depression among those suffering from MD. Indeed, most of the prior studies of cognitive vulnerability to depression were done in non-clinical populations, especially college students. Furthermore, these studies have overwhelmingly been conducted in North American and European samples. In our study, using a population of Han Chinese women with recurrent MD, we compared those who did with those who did not endorse the symptoms of the cognitive trio (worthlessness, hopelessness and helplessness) during their lifetime worst depressive episode, and asked whether these patients differed on other depressive symptoms, patterns of co-morbidity, features of course of illness and commonly assessed risk factors. Our goal is largely an exploratory one although from the prior clinical literature, we predicted that, among subjects with MD, endorsement of symptoms of the cognitive trio would be correlated with clinical severity and specifically with suicidal thoughts and plans.

## Method

### Study subjects

The data for the present study were drawn from the ongoing China, Oxford and VCU Experimental Research on Genetic Epidemiology (CONVERGE) study of MD. These analyses were based on a total of 1970 cases recruited from 51 provincial mental health centers and psychiatric departments of general medical hospitals in 40 cities in 21 provinces.

All cases were female and had four Han Chinese grandparents. Cases were excluded if they had a pre-existing history of bipolar disorder, any type of psychosis or mental retardation. Cases were aged between 30 and 60 years, had two or more episodes of MD, with the first episode occurring between the age of 14 and 50 years and had not abused drug or alcohol before the first episode of MD. The mean age was 45.1 (s.d. = 8.8) years.

All cases were interviewed using a computerized assessment system, which lasted on average 2 h. All interviewers were trained by the CONVERGE team for a minimum of 1 week in the use of the interview. The interview includes assessment of psychopathology, demographic and personal characteristics, and psychosocial functioning. Interviews were tape-recorded and a proportion of them was listened to by the trained editors who provided feedback on the quality of the interviews.

The study protocol was approved centrally by the Ethical Review Board of Oxford University and the ethics committees in participating hospitals in China.

### Measures

The diagnoses of depressive (Dysthymia, MD and Melancholia) and anxiety disorders [generalized anxiety disorder (GAD) and panic disorder] were established with the Composite International Diagnostic Interview (CIDI; WHO lifetime version 2.1; Chinese version), which classifies diagnoses according to the Diagnostic and Statistical Manual of Mental Disorders, 4th edition (DSM-IV) criteria.

Phobias, divided into five subtypes (animal, situational, social, and blood-injury, and agoraphobia) were diagnosed using an adaptation of DSM-III criteria requiring one or more unreasonable fears, including fears of different animals, social phobia and agoraphobia that objectively interfered with the respondent's life. The section on the assessment of phobias was translated by the CONVERGE team from the interview used in the Virginia Adult Twin Study of Psychiatric and Substance Use Disorders (VATSPUD) (Kendler & Prescott, 2006).

Additional information using instruments employed from VATSPSUD, translated and reviewed for accuracy by members of the CONVERGE team, was collected on premenstrual syndrome (PMS), postnatal depression (PD), parent–child relationship, SLEs, childhood sexual abuse (CSA) and neuroticism.

Information on PD was assessed using an adaptation of the Edinburgh Scale (Cox *et al.* 1987). Parent–child relationship was measured with the 16-item Parental Bonding Instrument (PBI) modified by Kendler (1996) based on Parker *et al.*'s (1979) original 25-item instrument. Three factors were extracted from these 16 items and labeled: coldness, protectiveness and authoritarianism. This three-factor PBI solution was found in this sample as well as by Kendler & Prescott (1996) in Virginia twins and has been further replicated in two other US samples (Cox *et al.* 2000; Lizardi & Klein, 2002), as well as in Japan (Sato *et al.* 1999) and Brazil (Terra *et al.* 2009). The SLEs section, also developed for VATSPSUD, assessed 16 traumatic lifetime events and the age at their occurrence. The CSA was a shortened version of the detailed module used in the VATSPSUD study, which was in turn based on the instrument developed by Martin *et al.* (1993). Neuroticism was measured with the 23-item Eysenck Personality Questionnaire (Eysenck & Eysenck, 1975), which was also an established instrument for measuring neuroticism.

The interview (named SysQ) was fully computerized into a bilingual system of Mandarin and English developed in-house in Oxford, UK. Skip patterns were built into SysQ. Interviews were administered by trained interviewers and entered offline in real time onto SysQ, which was installed in the laptops. Once an interview was completed, a backup file containing all the previously entered interview data could be generated with database-compatible format. The backup file together with an audio recording of the entire interview was uploaded to a designated server currently maintained in Beijing by a service provider. All the uploaded files in the Beijing server were then transferred to an Oxford server quarterly.

We assessed the three key cognitive symptoms – making up the cognitive trio – through the following questions in the MD section. During your worst episode: (1) Did you feel worthless nearly every day for 2 weeks or longer? (2) Did you feel hopeless about things? (3) Did you feel helpless much of the time? All the other depressive symptoms were also assessed at personal interview with the patient describing their lifetime worst episode of MD.

### Statistical analysis

All statistical analyses were carried out in SAS version 9.3 (SAS Institute Inc., USA). A χ^2^ test and tetrachoric correlations were used to investigate the association between the three cognitive symptoms of worthlessness, hopelessness and helplessness. We examined the relationship between categorical variables and cognitive symptoms using simple and multiple logistic regression. Stepwise logistic regression was performed to find the best predictors of the cognitive trio. Linear regression was performed to examine the relationship between continuous variables and cognitive symptoms. Estimates of odds ratios (ORs), β coefficients and their associated 95% confidence intervals (CIs) were derived. A biserial correlation test was used to examine the relationship between individual cognitive symptoms and neuroticism.

We first examined the relationship between the cognitive trio and other MD symptoms and features. The independent variables were the three cognitive symptoms and their total score on a 0–3 scale. The dependent variables included the presence of individual DSM-IV symptomatic (or A) criteria, four separate suicidal items (that is, thoughts of death, suicidal ideation, suicidal plan, and attempt suicide), the sum of DSM-IV A criteria that were met, age at onset, number of episodes, two depressive subtypes (Melancholia and PD), and a range of co-morbid disorders (dysthymia, GAD, panic disorder, and five subtypes of phobia).

To examine the predictors for the cognitive trio, we examined factors including CSA, parent–child relationship according to PBI, total SLEs, neuroticism and PMS.

## Results

### Clinical characteristics of MD patients

Of all the 1970 women with a history of recurrent MD, 81.3% met criteria for melancholia, 10.1% for panic disorder, 30.0% for GAD, 18.1% for dysthymia, and 20.3% had co-morbid PD. Agoraphobia was reported by 26.7% of the patients, social phobia by 31.8%, animal phobia by 55.6% and situational and blood injury phobia by 39.6% and 40.1%, respectively. CSA was reported by 9.8% of the patients and 72% reported the occurrence of one or more SLEs over their lifetime.

### Characteristics of the cognitive trio

During their worst episode of lifetime MD, worthlessness was reported by 79.6% of the patients, while hopelessness and helplessness were reported by 79.7% and 88.2% of the patients, respectively. When summarizing the total score of the cognitive trio, 66.0% of the patients experienced all three symptoms, 19.4% reported two symptoms, 10.9% experienced one symptom, and only 3.6% of the patients reported no symptoms. The three cognitive symptoms were all significantly associated with each other (*p* < 0.0001). The tetrachoric correlations (and ORs) were: +0.60 between worthlessness and hopelessness (OR 7.30, 95% CI 5.38–9.92), +0.46 between worthlessness and helplessness (OR 4.13, 95% CI 2.94–5.80), and +0.54 between hopelessness and helplessness (OR 6.06, 95% CI 4.53–8.11).

### Relationship between cognitive symptoms and MD symptoms

We examined the nine DSM-IV symptomatic (or A) criteria for MD separately with the three cognitive symptoms and then with their total score (Table 1). Worthlessness was strongly associated with the other eight criteria, with ORs ranging from 2.38 with insomnia to 6.10 with trouble concentrating. Except for insomnia, hopelessness was also statistically associated with all symptomatic criteria, with ORs varying between 1.56 (weight change) and 9.43 (thoughts of death). Helplessness had the weakest overall associations. It was significantly related to six criteria but not with loss of interest, weight change and insomnia.

**Table 1.** Relationships between the cognitive trio and other symptoms and clinical features of major depression

Variable	Worthlessness		Hopelessness		Helplessness		Total score	
	OR/β	(95% CI)	OR/β	(95% CI)	OR/β	(95% CI)	OR/β	(95% CI)
DSM-IV A criteria								
Depressed mood	5.29***	(1.82–15.33)	4.65**	(1.55–13.90)	6.56***	(2.19–19.70)	2.65***	(1.62–4.32)
Loss of interest	5.54^#^	(2.93–10.48)	4.56^#^	(2.35–8.86)	1.21	(0.47–3.15)	2.03^#^	(1.50–2.75)
Weight change	2.50^#^	(1.80–3.47)	1.56*	(1.09–2.21)	1.35	(0.87–2.10)	1.42^#^	(1.21–1.67)
Insomnia	2.38^#^	(1.53–3.71)	1.44	(0.88–2.35)	1.34	(0.73–2.45)	1.36**	(1.09–1.70)
Slowed down/restless	3.76^#^	(2.78–5.09)	2.24^#^	(1.64–3.08)	2.47^#^	(1.71–3.56)	1.85^#^	(1.59–2.14)
Fatigue	2.95^#^	(2.06–4.22)	1.89***	(1.29–2.78)	2.01**	(1.29–3.15)	1.62^#^	(1.36–1.93)
Worthlessness	–	–	7.30^#^	(5.38–9.92)	4.13^#^	(2.94–5.80)	–	–
Trouble concentrating	6.10^#^	(3.50–10.63)	2.96***	(1.67–5.24)	3.37^#^	(1.81–6.28)	2.21^#^	(1.71–2.86)
Thoughts of death	5.62^#^	(4.42–7.13)	9.43^#^	(7.36–12.09)	3.39^#^	(2.54–4.51)	3.32^#^	(2.90–3.81)
Number of MD symptoms	13.93^#^	(11.00–17.63)	6.47^#^	(5.21–8.04)	3.46^#^	(2.68–4.47)	3.64^#^	(3.24–4.08)
Death thoughts	5.88^#^	(4.64–7.44)	8.94^#^	(6.98–11.44)	3.57^#^	(2.69–4.74)	3.42^#^	(2.99–3.92)
Suicidal ideation	5.36^#^	(4.22–6.83)	9.52^#^	(7.30–12.42)	3.49^#^	(2.61–4.67)	3.44^#^	(2.99–3.97)
Suicidal plan	4.42^#^	(3.35–5.82)	8.47^#^	(6.08–11.81)	2.48^#^	(1.81–3.40)	2.90^#^	(2.48–3.39)
Suicidal attempt	5.39^#^	(3.51–8.27)	9.04^#^	(5.33–15.34)	2.87^#^	(1.82–4.52)	3.23^#^	(2.54–4.11)
Melancholia	3.89^#^	(3.02–5.02)	2.72^#^	(2.10–3.53)	2.21^#^	(1.62–3.03)	1.94^#^	(1.71–2.21)
Postnatal depression	1.09	(0.81–1.46)	1.44*	(1.05–1.96)	1.86**	(1.21–2.86)	1.21*	(1.04–1.40)
Dysthymia	1.28	(0.95–1.74)	2.35^#^	(1.64–3.36)	2.19***	(1.39–3.45)	1.46^#^	(1.23–1.72)
GAD	2.19^#^	(1.66–2.89)	2.25^#^	(1.70–2.99)	2.33^#^	(1.62–3.37)	1.68^#^	(1.46–1.94)
Panic	1.72*	(1.12–2.64)	3.02^#^	(1.79–5.11)	2.88**	(1.45–5.71)	1.76^#^	(1.37–2.27)
Phobia								
Agoraphobia	1.89***	(1.30–2.73)	1.58*	(1.11–2.25)	1.62*	(1.02–2.57)	1.40***	(1.17–1.67)
Social phobia	2.25^#^	(1.51–3.34)	2.22^#^	(1.49–3.30)	2.23**	(1.32–3.78)	1.69^#^	(1.38–2.08)
Animal phobia	1.33*	(1.03–1.72)	1.55**	(1.18–2.02)	1.60**	(1.13–2.26)	1.27***	(1.12–1.45)
Situational phobia	1.55**	(1.14–2.11)	1.66**	(1.22–2.26)	1.53*	(1.03–2.27)	1.33***	(1.14–1.56)
Blood injury phobia	1.51**	(1.13–2.04)	1.44*	(1.07–1.93)	1.60*	(1.09–2.36)	1.29***	(1.11–1.49)
Age at onset	−0.05	(−0.16 to 0.06)	−0.25^#^	(−0.36 to 0.14)	−0.19**	(−0.33 to −0.05)	−0.10***	(−0.15 to −0.05)
Recurrence	0.14*	(0.03–0.26)	0.10	(−0.01 to 0.21)	0.05	(−0.09 to 0.19)	0.07*	(0.01–0.12)

Data are given as odds ratio or β coefficient (95% confidence interval) for the effect of worthlessness, hopelessness, helplessness and their total score on other symptoms and clinical features of MD. Continuous variables were standardized before analysis. Odds ratios greater than 1 indicate that the presence of the specific component of the cognitive trio or their concurrence is more common in patients with the clinical feature or symptom.

DSM-IV, Diagnostic and Statistical Manual of Mental Disorders, Fourth Edition; MD, major depression; GAD, generalized anxiety disorder.

* *p* < 0.05, ** *p* < 0.01, *** *p* < 0.001, ^#^*p* < 0.0001.

We examined the association between the eight DSM-IV symptoms (worthlessness was excluded from consideration) and the combined cognitive symptoms. All associations were statistically significant, with ORs ranging from 1.36 with insomnia to 3.32 with thoughts of death. Dividing these ORs into those higher and lower than 2, we see that four criteria (depressed mood, loss of interest, trouble concentrating, and thoughts of death) had an OR >2.0 with the combined cognitive symptoms, while four criteria (weight change, insomnia, slowed down/restless, and fatigue) had weaker associations.

We then considered whether the cognitive symptoms predicted the symptomatic severity of MD. As expected, the associations with the sum of DSM criteria endorsed were all statistically significant, with ORs ranging from 3.46 (helplessness) to 13.93 (worthlessness).

The total score was especially strongly related to all four items assessing suicidal symptomatology (ORs 2.90–3.44). When examining the individual components of the cognitive trio, hopelessness (ORs 8.47–9.52) was consistently much more strongly related to all suicidal items than were worthlessness (ORs 4.42–5.88) or helplessness (ORs 2.48–3.57) (Table 1). Multiple logistic models also showed that hopelessness had the strongest relationship with the four suicidal items (Table 2).

**Table 2.** Relationships between the cognitive trio and suicidal items in multivariable logistic regression models

Variable	Worthlessness		Hopelessness		Helplessness	
	OR	(95% CI)	OR	(95% CI)	OR	(95% CI)
Death thoughts	3.34^#^	(2.56–4.36)	5.92^#^	(4.54–7.72)	1.53*	(1.09–2.14)
Suicidal ideation	3.09^#^	(2.36–4.04)	6.51^#^	(4.91–8.62)	1.55*	(1.10–2.18)
Suicidal plan	2.71^#^	(2.02–3.65)	6.25^#^	(4.42–8.85)	1.14	(0.79–1.63)
Suicidal attempt	3.32^#^	(2.13–5.17)	6.14^#^	(3.57–10.57)	1.40	(0.86–2.28)

Data are given as odds ratio (95% confidence interval) for the effect of including all the three cognitive symptoms of worthlessness, hopelessness and helplessness in the multivariable logistic model on each of the four suicidal items of major depression. Odds ratios greater than 1 indicate that the presence of the specific component of the cognitive trio or their concurrence is more common in patients with the suicidal item.

* *p* < 0.05, ^#^*p* < 0.0001.

### Relationship between cognitive symptoms and clinical features of MD

Two of the cognitive trio (helplessness and hopelessness) and the total score were significantly associated with an early age of onset (β’s varying between −0.25 and −0.15). The relationship between recurrence and cognitive symptoms was relatively weak although significant with worthlessness (β = +0.14) and the total score (β = +0.07) (Table 1).

We examined the association with two MD subtypes: melancholia and PD (Table 1). Melancholia was significantly positively related to all three individual symptoms and their total score (ORs 1.94–3.89). Except for worthlessness, PD was statistically associated with hopelessness and helplessness (ORs 1.21–1.86).

We then explored the association between the cognitive trio and co-morbidity. We examined dysthymia and a range of anxiety disorders (Table 1). Except for dysthymia with worthlessness, we found all the disorders were significantly positively associated with both the individual cognitive symptoms and their total score, with ORs varying between 1.27 (total score with animal phobia) and 3.02 (hopelessness with panic disorder). Phobias had the weakest overall association with the cognitive trio.

### Clinical predictors of cognitive trio

We next examined the predictors of the cognitive trio of symptoms both individually and their sum score. First, we examined one predictor at a time (Table 3), correcting for multiple testing (*p* < 0.005 correcting for 10 tests). Maternal and paternal protectiveness, total SLEs, neuroticism and PMS predicted risk for the sum of the cognitive trio. There were at least two significant predictors for each of the individual cognitive features (Table 3). We included all individually significant variables (*p* < 0.05) in stepwise logistic models to determine the best set of unique predictors for the cognitive symptoms. Maternal protectiveness and neuroticism uniquely predicted worthlessness. Paternal protectiveness, total SLEs and neuroticism predicted hopelessness. Neuroticism best predicted a greater risk for helplessness. Maternal protectiveness and neuroticism predicted the total cognitive symptom count (Table 4).

**Table 3.** Prediction of the cognitive trio

Predictor	Worthlessness		Hopelessness		Helplessness		Total score	
	OR	(95% CI)	OR	(95% CI)	OR	(95% CI)	OR	(95% CI)
CSA	1.30	(0.87–1.95)	1.62*	(1.05–2.49)	2.47**	(1.29–4.76)	1.60**	(1.14–2.26)
PBI								
Coldness from mother	1.00	(0.89–1.13)	1.08	(0.96–1.22)	1.05	(0.90–1.23)	1.05	(0.95–1.16)
Authoritarianism from mother	1.10	(0.97–1.24)	1.08	(0.96–1.22)	1.07	(0.92–1.25)	1.07	(0.97–1.18)
Protectiveness from mother	1.21***	(1.06–1.37)	1.20**	(1.06–1.36)	1.25**	(1.06–1.47)	1.23***	(1.11–1.36)
Coldness from father	1.07	(0.95–1.21)	1.16*	(1.02–1.31)	1.10	(0.94–1.28)	1.11	(1.00–1.23)
Authoritarianism from father	1.13	(1.00–1.27)	1.07	(0.95–1.21)	1.09	(0.93–1.27)	1.09	(0.99–1.21)
Protectiveness from father	1.17*	(1.03–1.33)	1.22***	(1.07–1.38)	1.12	(0.96–1.32)	1.20***	(1.08–1.33)
SLEs	1.23***	(1.08–1.41)	1.45^#^	(1.26–1.68)	1.26**	(1.06–1.50)	1.31^#^	(1.18–1.47)
Neuroticism	1.70^#^	(1.51–1.90)	1.86^#^	(1.66–2.10)	2.00^#^	(1.73–2.33)	1.90^#^	(1.73–2.10)
PMS	1.17**	(1.04–1.31)	1.28^#^	(1.14–1.45)	1.38***	(1.17–1.62)	1.25^#^	(1.13–1.38)

Data are given as odds ratio (95% confidence interval) for the effect of risk factors on the cognitive trio of major depression. A *p* value less than 0.005 indicates statistical significance after correcting for multiple testing. Odds ratios greater than 1 indicate that the factor is more common in patients with the presence of one specific component of the cognitive trio or their total score. Continuous variables were standardized before analysis so that the odds ratio represents the increase in risk for the cognitive trio associated with an increase of 1 standard deviation in the relevant variable.

CSA, Childhood sexual abuse; PBI, Parental Bonding Instrument; SLEs, stressful life events; PMS, premenstrual syndrome.

* *p* < 0.05, ** *p* < 0.01, *** *p* < 0.005, ^#^*p* < 0.0001.

**Table 4.** Prediction of the cognitive trio in stepwise logistic regression models

Component	Parameter	OR	95% CI
Worthlessness	Protection from mother	1.17*	(1.02–1.36)
	Neuroticism	1.56^#^	(1.36–1.79)
Hopelessness	Protection from father	1.16*	(1.00–1.33)
	Stressful life events	1.35**	(1.12–1.62)
	Neuroticism	1.64^#^	(1.42–1.90)
Helplessness	Neuroticism	2.01^#^	(1.68–2.40)
Total score	Protection from mother	1.17**	(1.04–1.32)
	Neuroticism	1.76^#^	(1.56–1.98)

Data are given as odds ratio (95% confidence interval) for the effect of including all significant predictors (*p* < 0.05) obtained from Table 3 in the stepwise logistic model on worthlessness, hopelessness, helplessness and their total score. Odds ratios greater than 1 indicate that the factor is more common in patients with the presence of one specific of the cognitive trio or their total score. Continuous variables were standardized before analysis so that the odds ratio represents the increase in risk for the cognitive trio associated with an increase of 1 standard deviation in the relevant variable.

* *p* < 0.05, ** *p* < 0.01, ^#^*p* < 0.0001.

Neuroticism was the only risk factor that significantly and uniquely predicted each of the three cognitive symptoms and their total score. Therefore, we examined the biserial correlation between neuroticism and the three symptoms. The biserial correlations with neuroticism were +0.30 (worthlessness), +0.35 (hopelessness) and +0.36 (helplessness).

## Discussion

Our study had two goals. First, we examined the association between the cognitive trio and the clinical symptoms and associated features of MD in a large clinically ill sample of Han Chinese women. There were two major findings. As predicted, the patients' cognitive trio was strongly associated with the severity of their depressive illness. Compared with those with no cognitive symptoms, those with all three symptoms during their worst depressive episode experienced a greater number of DSM-IV A criteria, more individual depressive symptoms, an earlier age at onset, a greater number of episodes, and more depressive subtypes and co-morbidities. Furthermore, the cognitive trio was more strongly associated with other cognitive than with the ‘biological’ or neurovegetative depressive criteria. The second goal of this study was to identify factors that were associated with the cognitive trio. Four major findings were noteworthy. First, the personality trait of neuroticism was strongly associated with an increased risk for the cognitive trio. Second, total SLEs predicted the cognitive symptom of hopelessness. Third, maternal and paternal protectiveness during the patients' childhood increased the risk for worthlessness, hopelessness and the total cognitive trio. Fourth, surprisingly, CSA did not show any unique association with the cognitive trio.

With regard to findings relevant to our first goal, our results were consistent with several prior investigations. Previous studies (Alloy *et al.* 1992, 2000; Haeffel *et al.* 2003) found that negative cognitive styles were associated with a greater likelihood of past major, minor, and hopelessness depression (HD). In a 2.5-year longitudinal study of college students with prior depression, Iacoviello *et al.* (2006) found that individuals with negative cognitive style experienced more depressive episodes with increased severity and more chronic depressions than those without this style.

The results of prospective studies from the Temple-Wisconsin Cognitive Vulnerability to Depression (CVD) Project (Alloy *et al.* 1999) suggested that in college students negative cognitive styles were more strongly predictive of increases in HD symptoms than non-HD depressive symptoms or symptoms of other types of psychopathology (Alloy *et al.* 1997; Metalsky & Joiner, 1997; Alloy & Clements, 1998; Hankin *et al.* 2001; Joiner *et al.* 2001). Partially similar to these results, our study found that hopelessness was significantly related to nearly all depressive symptoms hypothesized to be part of the HD symptom cluster (except insomnia). However, associations with hopelessness were no stronger than those with worthlessness or helplessness except for suicidal symptoms. Nevertheless, it is necessary to keep in mind that the CVD Project is a longitudinal study of college students while the CONVERGE Study is a cross-sectional study of patients with recurrent MD.

Our study provided strong evidence that the presence of any component of the cognitive trio during the worst depressive episode was predictive of melancholia. Previous work by Kendler (1997) suggested that melancholic MD is more severe than, but not etiologically distinct from, non-melancholic MD. Prior studies are mixed with respect to the causal relationship between cognitive features and melancholia. CBT was found to be effective for melancholia (Thase *et al.* 1991). However, the results of the CVD Project (Ingram *et al.* 2006) found that cognitive risk predicted first onsets of recurrences of HD, but not melancholic depression.

Findings from our second goal can also be corroborated elsewhere. Neuroticism has been reported to affect mood through cognitions (Nolan *et al.* 1998). Wilkinson & Blackburn (1981) found that the cognitive measures relating to Beck's theory of depression correlated highly with levels of neuroticism and depression. When levels of neuroticism and depression were controlled for, the cognitive differences between the depressed group, recovered depressed and control groups disappeared. Similarly, our findings indicate that both the separate components and the total cognitive trio are significantly but moderately related to the increased level of neuroticism.

Although our findings could not interpret the effect of the interaction between depressogenic schemata and negative life events on the cause of depression because of the lack of information of patients’ cognitive vulnerability, we do find a significant relationship between the cognitive trio and SLEs and CSA. These findings partially replicate those found in previous studies. According to Beck's (1967) theory, when confronted with a negative life event, individuals with negative cognitive style develop biased perceptions of themselves, their personal world, and their future. Rose & Abramson (1992) proposed that childhood negative life events, especially childhood maltreatment, may lead to the development of negative cognitive style. Evidence was found to support this hypothesis (Alloy *et al.* 2006*a*). In the CVD Project, participants’ cognitive risk status and hopelessness fully or partially mediated the relationship between levels of childhood emotional maltreatment and MD during the first 2.5 years of follow-up (Gibb *et al.* 2001*a*, *b*).

The association between the parent–child relationship and the cognitive trio found in our study indicate that parental protectiveness is specifically related to the total cognitive trio and the key components of worthlessness and hopelessness. However, our findings were inconsistent with at least one study in a Western college population. The results from the CVD Project suggested that parenting characterized by lack of warmth and caring, and by negative psychological control (e.g. criticism, intrusiveness, and guilt induction) was associated with depression and negative cognitive styles in offspring (Alloy *et al.* 2006*a*). Interestingly, while high levels of protectiveness were typically associated in Western samples with increased risk for MD (e.g. Kendler *et al.* 2000), in the CONVERGE sample, high levels of protectiveness from fathers were associated with a lower risk for MD (Gao *et al.* 2012). Further investigations are clearly warranted regarding the association between parenting style and cognitive symptoms of depression.

One of our most striking findings – the strong relationship between suicide and cognitive symptoms of depression – has important clinical implications. Consistent with both the Hopelessness Scale (Abramson *et al.* 1989) and Beck's theories of depression, we found a strong relationship between the cognitive trio and the MD A criterion of thoughts of death. Given this relationship, we examined the association between the cognitive trio and four items separately assessing thoughts of death, suicidal ideation, suicidal plan, and attempt suicide. Hopelessness was consistently the strongest predictor for all these items, providing strong support for the hypothesis that hopelessness is an especially strong predictor of suicide, the worst outcome for depressed individuals (Schueller & Seligman, 2008). Beck *et al.* (1985) found that the Hopelessness Scale and the pessimism item of the Beck Depression Inventory were factors that specifically predicted suicides. Other studies (e.g. Kazdin *et al.* 1983; Hill *et al.* 1988; Beck *et al.* 1993; Abramson *et al.* 1998) have also demonstrated a robust association between hopelessness and a range of suicidal symptomatology in MD patients of varying ages. Wenzel & Beck (2008) constructed a cognitive model of suicidal behavior in which hopelessness played a central role. Our results indicated that depressed patients with symptoms of the cognitive trio, especially hopelessness, should be carefully evaluated for suicide risk.

Finally, concerns have been raised about the comparability of MD in China *versus* in Western countries (e.g. Kleinman, 2004). Our findings suggest that as seen in both student and clinical populations in Western countries, symptoms of the cognitive trio also play a critical role in the psychopathology of depression in Han Chinese women.

### Limitations

These findings should be interpreted in the light of two important methodological limitations. First, our study was carried out solely with Han Chinese women with recurrent MD identified through hospitals. Our findings may or may not generalize to Han Chinese males or Chinese females who do not seek treatment. Moreover, we are not able to comment on the extent to which our findings will apply to those with a single depressive episode. Second, because of the retrospective nature of our data, we cannot determine the degree to which the associations between the cognitive trio and features of MD and risk factors are causal or a result of retrospective recall bias, or other unknown confounding factors. As prior studies have suggested, some of the clinical features of MD are recalled with only moderate reliability (Bromet *et al.* 1986; Bradbum *et al.* 1987).
